# Role of Bioadsorbents in Reducing Toxic Metals

**DOI:** 10.1155/2016/4369604

**Published:** 2016-12-19

**Authors:** Blessy Baby Mathew, Monisha Jaishankar, Vinai George Biju, Krishnamurthy Nideghatta Beeregowda

**Affiliations:** ^1^Department of Biotechnology, Sapthagiri College of Engineering, 14/5 Chikkasandra, Hesarghatta Main Road, Bangalore, Karnataka 560057, India; ^2^CUFE, Christ University, Kanmanike, Kumbalgodu, Bangalore, Karnataka 560074, India

## Abstract

Industrialization and urbanization have led to the release of increasing amounts of heavy metals into the environment. Metal ion contamination of drinking water and waste water is a serious ongoing problem especially with high toxic metals such as lead and cadmium and less toxic metals such as copper and zinc. Several biological materials have attracted many researchers and scientists as they offer both cheap and effective removal of heavy metals from waste water. Therefore it is urgent to study and explore all possible sources of agrobased inexpensive adsorbents for their feasibility in the removal of heavy metals. The objective was to study inexpensive adsorbents like various agricultural wastes such as sugarcane bagasse, rice husk, oil palm shell, coconut shell, and coconut husk in eliminating heavy metals from waste water and their utilization possibilities based on our research and literature survey. It also shows the significance of developing and evaluating new potential biosorbents in the near future with higher adsorption capacity and greater reusable options.

## 1. Introduction

In the last century many products such as medicines, disinfectants, laundry detergents, paints, surfactants, pesticides, dyes, preservatives, personal care products, and food additives have been found to be threatening to human as well as the environment [1, 2]. Various industries like fuel production units, atomic energy stations, electroplating and fertilizer industry, leather and electrical appliance manufactory, and iron enterprises generate enormous wastes containing large amount of toxic heavy metals discarded into the environment resulting in ecological imbalance. The pollutants and decaying organic matter in waste water take up the dissolved oxygen and excessive nutrients like phosphorus and nitrogen cause eutrophication which promotes excessive plant growth and reduces available oxygen in the water body. Bacteria, viruses, and disease-causing pathogens also pollute beaches and contaminate shellfish populations, leading to restrictions on human recreation and drinking water consumption. Metabolism dependent and independent processes can also result in the accumulation of large amount of metals [3] and trigger the free radical response leading to oxidative stress [4].

### 1.1. Biosorption

**Figure 1:**
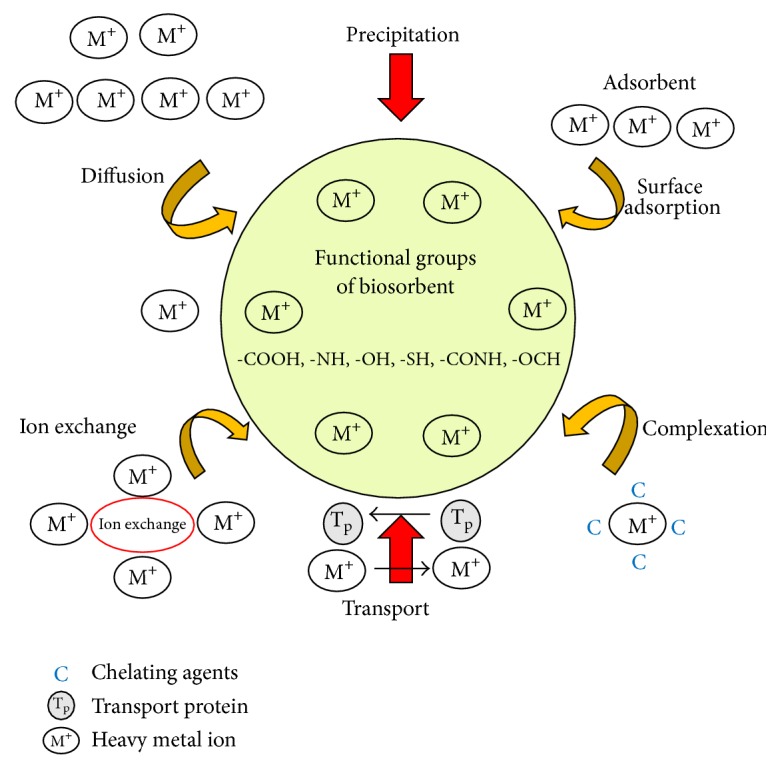
Biosorbent mechanisms.

The removal of metals or nonmetals and tiny particulates from a solution by means of any biological component is known as biosorption [5]. Cellular products and living and nonliving biomass can be used for effective adsorption [3], but their cost-effectiveness and reusability factor still remains under question. There are various physical, chemical, and biological methods to remove metal ions from aqueous solutions. Some of the conventional techniques like filtration, membrane technology, and ion exchange are very expensive and chemical precipitation and electrochemical treatment prove to be ineffective especially when the concentration of metal ion is 1–100 mg/L. It also results in large sludge production [6]. Many biological materials have high eradication rate in decreasing the concentration of heavy metals from ppm to ppb level [3]. Few types of biosorbents bind onto heavy metals with no specific priority, whereas others are specific for certain types of metals [7, 8]. This is one of the major reasons that research and studies on biosorption have become one of the most active work areas. In particular the low cost biosorbents obtained from agricultural and animal wastes are of major interest [9]. But the selection of biosorbent for a specific metal is a challenge and merely a hit and trial method, resulting from a series of experiments and in-depth research. We have attempted to carry out relevant researches on biosorption phenomena for the effective removal of metal ions using various agricultural wastes and to provide a summary of available information on a wide range of biosorbents through this paper as shown in Figure 1 [10].

### 1.2. Adsorption

A separation where in certain fluid elements are transferred to solid surface of the adsorbents is known as adsorption. Transfer of molecules from bulk solution to solid surface occurs based on concentration gradient. Here when a solid surface is exposed to a fluid phase, the molecules from the fluid phase accumulate or concentrate at the surface of a solid. All adsorption processes depend on mass transfer rates and solid-liquid equilibria [31]. The process is referred to as “desorption” if the mass transfer takes place in opposite direction. Highly porous materials are chosen as adsorbents, and adsorption occurs mostly on the pore walls or at particular sites within the particle. Difference in shape, molecular weight, or polarity makes the molecule stronger on the surface of other materials which makes separation easier. Solute diffusion rate in the capillary pores of adsorbent determines the overall adsorption rate. Rate of adsorption is equal to the square root of contact time with adsorbent.

#### 1.2.1. Types of Adsorption Processes

Adsorption can be carried out as batch, semibatch, and continuous processes. When little quantities are to be treated, batch processes are generally carried out and the equilibrium distribution depends on the contact time in batch process [32]. Heavy metals in waste water include Cd, Cu, Zn, Cr, and Ni and most of the companies fail to solve the problems caused due to these discharge, as they lack proper technical knowledge and economic capabilities. The concentration of various heavy metals in waste water ranges from 10 ppm to 100 ppm [33]. Adsorbate and adsorbent experience certain attractive forces which bind them and these forces can be due to Van der Waals forces which are weak in nature or they may be due to chemical bonds which are strong in nature. On the basis of attraction and the strength of force prevailing between adsorbate and adsorbent, adsorption can be categorized into two. 

**Figure 2:**
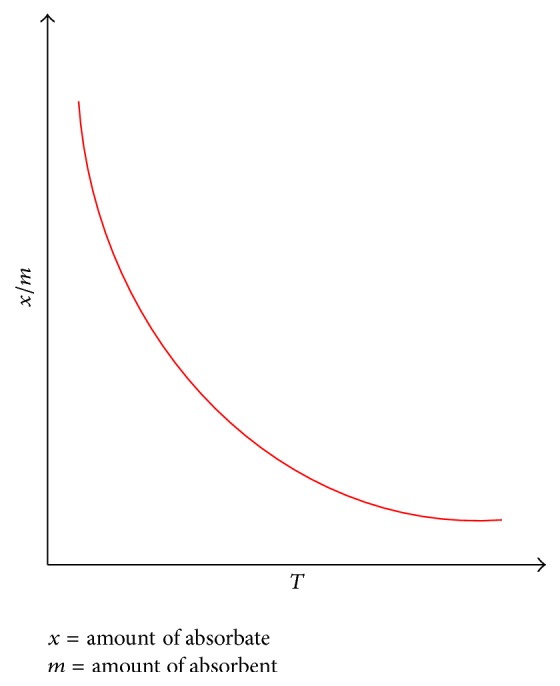
Physical adsorption versus temperature.

*(1) Physical Adsorption*. Also known as physisorption, it occurs when the attractive forces present between adsorbate and adsorbent are weak like Van der Waals forces as in Figure 2. It has low enthalpy of adsorption (i.e., Δ*H*_adsorption_ = 20 to 40 KJ/mol) and occurs with development of multilayer of adsorbate on adsorbent. This phenomenon decreases with an increase in temperature and usually takes place at a lower temperature which is generally much below the boiling point of the adsorbate [34].

**Figure 3:**
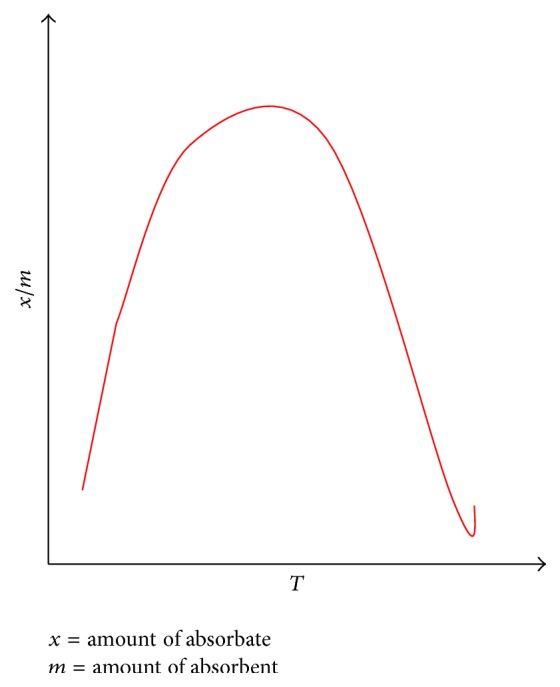
Chemical adsorption versus temperature.

*(2) Chemical Adsorption*. Also known as chemisorption, it occurs when the attractive forces between the adsorbate and adsorbent are chemical forces of attraction or via chemical bond as in Figure 3. Here only a single layer formation of the adsorbate on adsorbent takes place and it has a high enthalpy of adsorption (i.e., Δ*H*_adsorption_ = 200 to 400 KJ/mol). This phenomenon first increases and then decreases with a rise in temperature [35].

### 1.3. Factors Affecting the Adsorption Process

Over the past 20 years there has been an exponential growth in the world's population. This has led to the environmental buildup of waste products, among which heavy metals are of major concern. Heavy metals can be biodegradable which are being added to soil, water, and air in escalating amounts as well as nonbiodegradable. Some metals like zinc, magnesium, and copper are required for animal and plant life as micronutrients but the same would become more hazardous if they are taken up by plants or animals in large amounts [36]. The various factors on which the adsorption process depends are as follows.

#### 1.3.1. Temperature

As the temperature increases, the adsorption capacity is found to decrease and vice versa. It is an exothermic process overall.

#### 1.3.2. pH

As pH increases from 7.0 to 7.5, the retention capacity of the adsorbing surface increased significantly, whereas in lower pH the adsorption process was affected.

#### 1.3.3. Pressure

With increase in pressure, adsorption increases up to a certain extent till saturation level is reached but after that no more adsorption takes place no matter how high the pressure is.

#### 1.3.4. Adsorbent Activation

To provide higher number of vacant sites on surface of adsorbent, this can be done by breaking solid crystal in small pieces, heating charcoal at high temperature, breaking lump of solid into powder, or other methods suitable for particular adsorbent.

#### 1.3.5. Surface Area of Adsorbent

As adsorption is a surface phenomenon it increases with increase in surface area. Thus for any big molecule with a higher surface area, the adsorption efficiency will increase. As we know, volume of an ideal gas at STP = 22.4 *L* = 22.4 dm^3^ and the number of gaseous molecules present at STP = 6.023*∗*10^23^ molecules. Let us assume that Vmono is the adsorbed volume of gas at high pressure conditions so as to cover the surface with a uniform layer of gaseous molecules. Then assuming the total number of adsorbed gas molecules as *N* corresponding to volume *V*_mono_, it can be given as

(1) $$\begin{array}{c} N=\left[\;\frac{V_{mono}}{22.4\;{\text{dm}}^{\text{3}}\;{mol}^{-1}}\;\right]\times 3.023\times 10^{23}\;{mol}^{-1}. \end{array}$$

### 1.4.  Problems Associated with the Existing Technologies

The technologies which are being used at present for removal of metal ion from waste water are very expensive. They include ion exchange resin, solvent extraction, electrolytic and precipitation processes, electrodialysis, and membrane technology [37]. There are wide ranges of conventional technologies from granular activated carbon to reverse osmosis. These processes are, however, not economically feasible for small scale industries prevalent in developing economies due to large capital investment [38]. The most widely used method for removing heavy metals from waste water is by precipitation process but it results in the production of sludge containing high levels of heavy metals. Hence to purify the effluent prior to discharge there is a need for additional treatments like ion exchange, reverse osmosis, or adsorption process [39].

## 2. Technologies That Exist to Remove Metal Ions from Effluents

The removal of metal ions from effluents can be achieved by many methods. The technologies are divided into three types, namely, physical, chemical, and biological. The physical and chemical processes form the principal method of waste water treatment in industries. There were many merits and demerits caused due to high cost and disposal problems.

### 2.1. Physical Methods

Membrane filtration and adsorption are the physical methods. Membrane filtration processes include nanofiltration, reverse osmosis, and electrodialysis. Membrane fouling forms the major disadvantage of this membrane filtration. Since proper design of the adsorption process will create high quality treated effluents, adsorption process can be regarded as the best method for heavy metal removal from effluents.

### 2.2. Chemical Methods

To achieve the desired water quality in the most economical way, processes of different combinations were used. Electroflotation, electrokinetic coagulation, coagulation combined with flotation and filtration, conventional oxidation methods by oxidizing agents, irradiation, and electrochemical processes are the techniques which fall under chemical methods [40]. These chemical technologies are having many disposal problems and are very costly too. Even though these methods are efficient for removing the contaminants from waste water, they are commercially unattractive and very expensive. Some of the common problems are high electrical energy demand and consumption of chemical reagents.

### 2.3. Biological Methods

**Figure 4:**
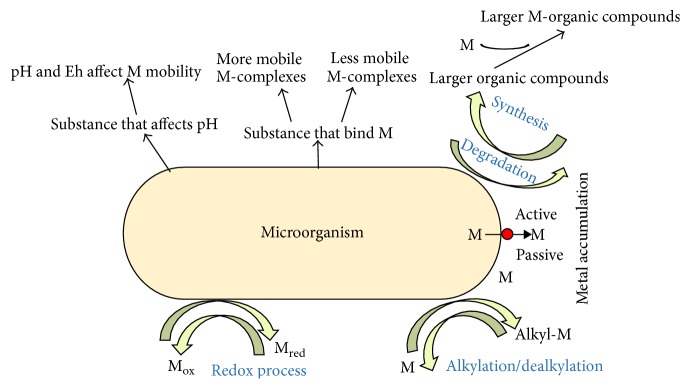
Metal accumulation process by microorganisms and its influence on metal mobility.

New separation methods are necessary to reduce heavy metal concentrations to environmentally tolerable levels at reasonable cost because most conventional methods are neither effective nor economical, especially when used for the reduction of heavy metal ions to low concentrations. This goal can be achieved through bioremoval [41–43]. The accumulation and concentration of heavy metals from aqueous solutions using biological materials is known as bioremoval [37] as shown in Figure 2 [44]. Many studies were done for the removal of heavy metals from solution by utilizing different agricultural products and byproducts. Several studies showed the efficacy of many organic waste components as sorbents for heavy metals [45–47]. If the adsorbent is inexpensive and does not require an additional pretreatment step before application, this process provides an outstanding alternative for treatment of contaminated water. But biological method is not favorable as it requires large area and has less design flexibility and lesser modes of operation and is constrained by sensitivity towards diurnal variation including certain chemical toxicity [48]. In contrast to other techniques adsorption has originated to be superior for water reuse in terms of primary cost, elasticity and straightforwardness of design, ease of operation, and insensitivity to toxic pollutants and there is no production of dangerous materials [49]. Other than the above listed methods, the one method which is from the nature itself which would not just remove the metal ions but is also an example of effective usage of biowaste is bioadsorption, that is, the adsorption of any constituents, like metals, onto the surface of biological components. Selecting the most capable types of biomass from an enormously huge pool of readily accessible and cheap biomaterials was the first most important challenge for the biosorption field [50]. Full scale biosorption process requires the biological materials which have high metal binding capabilities and specific heavy metal selectivity. Biosorption mechanisms are understood to a limited extent. It may involve one process or a blend of processes like adsorption, electrostatic interaction, chelation, microprecipitation, and ion exchange [3, 51, 52]. These biomasses have been tested and reported to bind a variety of heavy metals to different extents. Microbial biosorption is another aspect under this which is carried out by different organisms like bacteria, fungus, and algae. Nearly 50 microbial strains of microorganisms, capable of degrading xenobiotics, have been isolated, such as* Pseudomonas, Mycobacterium, Alcaligenes, and Nocardia*. Microbial degradation of heavy metals assumes significance, since it provides an effective and economic means of disposing of toxic chemicals, particularly the environmental pollutants (Figure 4).

## 3. The Green Technology: Usage of Effective Bioadsorbents

### 3.1. Wheat Bran

Wheat bran acts as an efficient adsorbent for the heavy metal ions removal such as Pb(II), Cu(II), and Cd(II). Another study stated that significant adsorption in copper ions could be achieved by pretreatment with strong dehydrating agent like sulphuric acid (H_2_SO_4_) as the micropores and macropores conversion leads to increase in adsorption [53]. The maximum capacity achieved for 30 min was 51.5 mg/L (at pH 5) for Cu(II) ions. This method when used for lead ions showed maximum removal up to 82.8% at pH 6 after 2 h of equilibrium time [54] and for cadmium ions 101 mg/L at pH 5, efficient adsorption was seen at a contact time of 4 hrs. Langmuir, Freundlich, and Redlich-Peterson isotherm models were used to determine the adsorption capacity of wheat bran out of which Redlich-Peterson was found to be the best fitted model for adsorption.

### 3.2. Rice Husk

One of the agricultural wastes generated especially in rice producing countries, like Asia, is rice husk. Around 500 million metric tons of rice is produced around the world out of which rice husk constitutes 10 to 20%. Dry rice husk consists of 70 to 85% of organic matter, mainly sugars, lignin, cellulose, and so forth. It contains cellulose (32.24%), hemicelluloses (21.34%), lignin (21.44%), and mineral ash (15.05%) as well as high percentage of silica in its mineral ash, which is approximately 96.34% [55, 56]. Rice husk is chemically stable, has high mechanical strength, and is insoluble in water which makes it one of the best adsorbents for heavy metal removal. The removal of heavy metals such as Cd, Pb, Zn, Cu, Co, Ni, and Au from rice husk was also studied [57]. Either modified or unmodified rice husk can be used for the treatment of heavy metals. Rice husk can be modified using hydrochloric acid or sodium carbonate [58] or by sodium hydroxide [59] or treated with epichlorohydrin [58]. Tartaric acid also can be used for modification [60, 61]. Pretreating rice husks helped in reducing cellulose crystallinity and rise in surface area or porosity and in removal of lignin and hemicelluloses by which adsorption capacity on heavy metals increased. Rice husk when treated with sodium carbonate and sodium carbonate or hydrochloric acid enhanced the cadmium adsorption capacity [58]. An adsorption site on the rice husk surface was protonated when it was treated with HCl and hence the heavy metals were left in the aqueous solution itself. The base soluble materials on rice husk were removed through NaOH treatment which also doubled the rate of cadmium adsorption (i.e., 7 mg/L) when compared to the rice husk adsorption of 4 mg/L. Some studies revealed that high reaction rate and improved cellulose hydrolysis can be achieved through pretreatment using dilute sulphuric acid [62]. Cellulose hydrolysis can be achieved through concentrated acids but they are corrosive and toxic, so they need to be recovered [63]. Another study showed the adsorption of copper and lead on rice husk modified using acids like citric acid, salicylic acid, tartaric acid, oxalic acid, mandelic acid, malic acid, and nitrilotriacetic acid [59]. Among these, rice husk heated with tartaric acid showed maximum adsorption. The effect of chelators on lead was also investigated which showed that when there was an increase in molar ratios of chelators like ethylenediaminetetraacetic acid and nitrilotriacetic acid there was a considerable raise in the adsorption of lead. Parameters like pH, initial concentration of adsorbent, particle size, and temperature affected the adsorption efficiency and it was found that modified rice husk had maximum capacity to adsorb copper and lead from aqueous solutions mainly when the pH was around 2-3 [60]. Rice husk treated with phosphate adsorbed maximum nickel and cadmium [64], whereas at a pH of 12, there was 90% adsorption of chromium when rice husk carbon was used as adsorbent [65]. This was done by carbonizing rice husk with sulphuric acid and then activating it by CO_2_ activation. 99% of hexavalent and 88% of total chromium were removed using this method. Rice husk showed 8.9 mg/g adsorption and commercial carbon showed 6.3 mg/g for chromium removal when column studies were done [66]. Another study using some of the dyes, Procion Red or Procion Yellow, showed that Procion Red dye treated rice husk removed 99.2% cadmium and 99.8% lead. Procion Yellow dye treated husk adsorbed 100% lead and 93.3% mercury. When waste water containing heavy metals was treated with rice husk it showed 79% chromium, 85% zinc, 80% copper, and 85% cadmium ions adsorption on the rice husk [67]. Another study with green algae as adsorbents resulted in 90% removal of heavy metals like Sr, Cd, Ni, Pb, Zn, Co, Cr, and As but could remove only 80% nickel [68]. Microporous and mesoporous activated carbon forms of rice husk were considered for the adsorption of chromium [69]. Rice husk was classified into two types and could be used as adsorbents on small waste water treatment plants and found adsorption about 100% for many heavy metals such as iron, manganese, zinc, copper, cadmium, and lead [70]. Raw rice husk was utilized for the removal of Cr(VI) and they concluded that an adsorbent dosage of 70 g/L for 2 hours at a pH 2 showed increase in the rate of adsorption and 66% of Cr(VI) was removed [71].

### 3.3. Sugarcane Bagasse

Sugar refining industry produces waste called bagasse pitch where the residual cane pulp is left over after sugar has been extracted. Cellulose, pentosan, and lignin are the components present in it [72]. The adsorption of cadmium and lead was studied on lignin obtained from sugarcane bagasse [73]. They found that ionic strength was inversely proportional to the adsorption capacity and reported that adsorption of lead followed Langmuir's model and temperature greater than 30°C worked best for cadmium removal. Lignin which was carboxymethylated had the ability to adsorb more amount of lead compared to cadmium. Temperature was found to be the most important as adsorption increased with increase in temperature. Single and multicomponent cadmium and zinc adsorption was done by using activated carbon prepared from bagasse [72], where a pH greater than 8 gave 100% adsorption of cadmium and zinc. 0.8 g/50 mL of adsorbent was necessary to remove 80%–100% of chromium at pH 1 but when they increased pH to 3, there was a decrease in adsorption efficacy by 15% [74]. According to another study the removal of hexavalent chromium from waste water using bagasse and coconut jute required low pH for maximum adsorption. Activated carbon obtained from jute had an adsorption efficiency of about 99.8% at pH 2. It was more stable at higher pH and hence it proved to be the most active adsorbent [75].

### 3.4. Fruit/Vegetable Waste

The fruit/vegetable wastes like banana and orange peels were modified using acid and alkali solutions. The adsorption of Cu2+, Zn2+, Co2+, Ni2+, and Pb2+ onto the modified fruit/vegetable wastes was studied [76] Lead was adsorbed to the maximum and cobalt to the least. Banana showed an adsorption capacity of 7.97 (Pb2+), 6.88 (Ni2+), 5.80 (Zn2+), 4.75 (Cu2+), and 2.55 (Co2+) mg/L, when compared to orange peels which showed 7.75 (Pb2+), 6.01 (Ni2+), 5.25 (Zn2+), 3.65 (Cu2+), and 1.82 mg/L (Co2+). It was shown that adsorption capacity of acid treated peels was more efficient than alkali or water treated peels. NaOH and Ca(OH)_2_ were used to regenerate the metal ions [77]. Saponified gels were prepared from orange peel constituents such as cellulose, hemicelluloses, pectin, limonene, and other lesser molecular weight compounds using Ca(OH)_2_. Two different types of saponified gels were prepared using Ca^+2^ form and H^+^ form and efficiency of both the saponified gels to remove heavy metals such as iron, lead, copper, zinc, cadmium, and manganese was analyzed. It was found that saponified gel prepared from Ca^+2^ can adsorb lead to the maximum compared to manganese and saponified gel prepared from H^+^ can adsorb metals in the order of lead > iron > copper > zinc > manganese. Increase in pH increased the heavy metal adsorption. But in the case of Fe^3+^ it was not the same as it formed soluble complexes such as Fe(OH)^+^, Fe(OH)_2_^+^, Fe(OH)_2_^4+^, and Fe(OH)_4_ at pH 3. Significantly the study of both types of gels infers that they act as effective adsorbent in acidic solutions because of ion exchange mechanism. Acid treated fruit wastes such as cornelian cherry, apricot stone, and almond shell were able to adsorb maximum Cr(VI) at pH 1 [78]. They found out that the lower the concentration of metal ions, the higher the rate of adsorption. Their studies also concluded that cornelian cherry required an equilibrium time of 20 h when chromium concentration was 53 mg/L but when the concentration was increased to 203 mg/L the equilibrium time also rose to 70 h. ZnCl_2_ treated olive stone showed maximum adsorption for cadmium and lead ions of about 85 mg/L [79], compared to untreated olive stone [80]. Citric acid treated orange peels showed maximum adsorption for cadmium. Cadmium adsorption increases due to the interaction between cellulose present in orange peels and citric acid as a result of the formation of ester linkage and introduction of carboxylic group [81]. Adsorption and recovery of cadmium from orange peels increase, when they are treated with acids of high concentration [82]. Agricultural byproducts such as sugarcane, bagasse, peanut shells, macadamia, nut hulls, rice hulls, cottonseed hulls, corn cob, soybean hulls, almond shells, almond hulls, pecan shells, English walnut shells, and black walnut shells were also treated with citric acids to increase adsorption of copper [83]. All these byproducts were treated with NaOH and then with citric acid to increase adsorption of heavy metals. Among all the byproducts, citric acid treated soya bean hulls showed maximum adsorption for copper whereas English walnut shells and black walnut shells showed least adsorption as they are high density materials and lignin present in them blocks the reactive sites to citric acid resulting in minimum adsorption of copper. Another study reported that citric acid treated peanut shells showed maximum adsorption for metal ions such as copper, cadmium, nickel, zinc, and lead when compared to unmodified peanut shells. Peanut shells were treated with NaOH first and then with citric acid to obtain more active binding sites for metal adsorption. NaOH removes tannins and deesterifies which increases its capacity to adsorb metal ions. Vegetable wastes such as carrot residues were treated with HCl to eliminate the tannins, resins, reducing sugars, and colored materials which in turn increase its capacity to remove metal ions such as chromium, copper, and zinc [84]. Kinetic study revealed that 70% of metal ions were adsorbed within 10 min and maximum adsorption of chromium (45.09 mg/L), copper (32.74 mg/L), and zinc (29.61 mg/L) was seen at higher pH values of 4 for chromium and 5 for both zinc and copper ions.

### 3.5. Soya Bean Hulls, Cottonseed Hulls, Rice Bran, and Straw

Soya beans were modified using citric acid and its adsorption efficiency for heavy metals. Copper ions were considered as typical metal ion for studying the adsorption of soybean hulls. 0.1 N NaOH was used to extract the hulls, and then it was modified at 120°C for 90 minutes using citric acid whose concentration ranged from 0.1 M to 1.2 M. Unmodified hulls showed an adsorption capacity of 0.39 m moles/g whereas citric acid modified hulls had an adsorption capacity of 0.68 to 2.44 m moles/g. Soya bean hulls when treated with NaOH and modified with 0.6 M citric acid could remove about 1.7 m moles of copper ions [85]. Reaction with citric acid showed an increase in carboxyl group on the hulls which in turn resulted in an increased uptake of copper ions. On the basis of dry weight, soya bean consisted of 109, 10.0, 36.4, 49.1, 676, 137, and <10 (mg/g) of protein, lipid ash, lignin, cellulose, hemicelluloses, and silica, respectively. Langmuir model was used to determine the adsorption efficiency. Soya bean and cottonseed hulls had higher adsorption efficacy when treated with NaOH and HCl when compared to water washed hulls [86]. When cotton seeds and soya bean hulls were heat treated they showed lower adsorption properties than water washed hulls. When the hulls were reprocessed after one adsorption/desorption cycle there was a decrease in adsorption capacity and so hulls were regarded as single use adsorbents.

### 3.6. Corncobs

Corncobs were initially activated by high temperature carbonization but a certain study revealed that corncobs can also be activated by chemical methods using acids for copper adsorption [87]. Corncobs were treated with acids containing functional groups such as -OH, -COOH, and -COO at 150°C which reduced the pH from 5.2 to 2.7 and a maximum adsorption of copper (31.45 mg g/L) at a pH value of 4.5 was seen. The copper adsorption by corncobs decreased to 53%, 27%, and 19% in presence of interfering ions like Pb(II), Ca(II), and Zn(II), respectively. Hydrogen peroxide can be used to regenerate those acid treated corncobs and almost 90% of copper can be recovered. Corncob oxidation using nitric and citric acids showed significant areas for adsorption [88].

### 3.7. Tree Barks

Hydrochloric acid treated barks mainly sal (*Shorea robusta*), mango (*Mangifera indica*), and jackfruit (*Artocarpus integrifolia*) were used in removal of copper from aqueous solutions. Untreated barks had lesser capacity to chelate when compared to modified barks. The advantage of pretreating the barks was the overcome of those organic compounds which gave color to metal ion containing solution. Sal bark, mango, and jackfruit showed an adsorption capacity of 51.4 mg g/L, 42.6 mg g/L, and 17.4 mg g/L, respectively, for copper ions. Successful recovery of adsorbent can be obtained using HCl in higher concentration [89].

### 3.8. Neem Bark

It was found that* Azadirachta indica* bark (neem bark) has maximum adsorption capacity towards iron metal ions. The bark has different functional groups at the surface of adsorption site which makes it an efficient adsorbent for iron. Maximum adsorption was seen within 30 minutes of contact time and later the reaction slowed down as it approached to steady state. When neem and babool tree barks were kept in solution containing many metal ions, 80–90% of chromium, cadmium, and manganese were adsorbed within a contact period of 10 minutes at pH 2 [90]. The eucalyptus bark removed 99% of chromium(VI) and neem bark removed zinc metal ion within an equilibrium adsorption time of 35 minutes [91]. Due to less number of active adsorbent sites, the adsorption decreases as the contact time attains steady state and hence the removal of metal ions using neem bark acts as a low cost method for the treatment of toxic water containing iron metal ion [92].

### 3.9. Almond Shells

The ability of activated carbon prepared from almond shell to eradicate iron present in the synthetic solution proved that the ability of activated carbon to remove iron was very high. In this study the contact time was kept around 20 minutes and it was observed that there was an increase in iron uptake. Later when the contact time was increased above 20 minutes, there was no change in the percentage uptake of iron. Thus results indicated that 20 minutes was the optimum adsorption time for iron uptake. pH also showed its effect on iron adsorption. Adsorption of iron present in synthetic water onto almond shells increased as the pH increased from 1 to 9. But iron removal was found to be highest at a pH value of 5 for almond shells [93].

### 3.10. Peanut Shell

Peanut shell biomasses were used for the study of biosorption of chromium ions and copper ions from aqueous solutions. Biosorption was studied as a function of temperature, pH, and initial concentration of the biomass. Metals were studied separately for optimum sorption conditions. Different kinetic models were used to study the experimental data. These studies revealed that affinity of peanut shell towards Cu(II) and Cr(III) ions was very high. The adsorption capacity of copper and chromium was found to be 25.39 mg and 27.86 mg per gram of peanut shell biomass, respectively. These results proved that peanut shell can be used as an efficient low cost bioadsorbent for removing heavy metals in waste water. The effects of pH and cadmium concentration on adsorption were also analyzed. Results of batch experiments showed an adsorptive capacity of 87.72 mg/g for peanut shells. To study the adsorption equilibrium of peanut shells Langmuir isotherm model and Freundlich isotherm model were used [94].

### 3.11. Green Coconut Shell

Coconut shell is a byproduct which is composed of 35–45% and 23–43% of lignin and cellulose, respectively [95]. It acts as a strong potential adsorbent for metal ion adsorption and also cellulose contains carboxylic and phenolic acids which are polar functional groups which help in metal binding [96, 97]. At lower to higher range of metal concentrations (20–1000 mg/L), coconut shell has a capacity to adsorb cadmium ions. The study was concluded for the best adsorption of cadmium metal ion at a pH value of 7. The adsorption data for cadmium ion obtained was studied using isotherm models such as Freundlich and Langmuir adsorption models at 27°C.

### 3.12. Tamarind Pod Shells

Tamarind pod shells can be economically recycled for the treatment of water containing chromium metal ions. The shells were washed with distilled water in order to remove the dirt and other coloring substances by boiling the pod shells at 105°C. The rate of adsorption for different concentrations of Cr(VI) metal ions was done by varying the factors like agitation time, adsorbent dosage, and pH. The adsorption data was well fitted into Langmuir and Freundlich isotherm models. There was maximum adsorption of chromium onto the tamarind shells at pH 2 whereas the rate of adsorption decreased as the pH value increased. Equilibrium time for adsorption was considered using different concentrations of chromium metal ion solution and the time attained was about 60 minutes. Desorption of metal ion from shells was studied using acid and alkali solutions. Binding of metal ion to shells must be possibly by chemisorptions or ion exchange which was inferred during the desorption studies of metal ion [98].

### 3.13. Egg Shells

Egg shells act as a good adsorbent for the removal of toxic heavy metals. The waste egg shells were calcinated in the pretreatment process for effective adsorption of heavy metals. These calcined egg shells were used in the removal of heavy metals from waste water. There was 100% adsorption of cadmium and 99% adsorption of chromium for a contact time of about 10 minutes. Even natural egg shell can be used as adsorbent but it is more efficient for the removal of lead compared to cadmium and chromium. There was less adsorption of cadmium and chromium even when the contact time was increased to 60 min as the rate of reaction was slow for natural egg shell. Cadmium and chromium showed a rapid adsorption rate, when the pH was raised from 6.55 to 12.0 within the contact time of 20 s. Calcined egg shell can be used to treat heavy metals found in the electroplating waste water [99].

### 3.14. Silverleaf Nightshade

Silverleaf nightshade* (Solanum elaeagnifolium)* was used as a biosorbent by base modification using NaOH for the removal of metal ions such as lead, copper, nickel, cadmium, zinc, and chromium by means of batch experiments studies. The adsorption capability of silverleaf nightshade for lead, copper, nickel, cadmium, zinc, and chromium(III) was determined by considering the parameters such as pH, equilibrium time, and metal binding capacity. At pH 5 within a time period of 10 to 15 minutes and at pH 2 there was optimal adsorption of chromium(VI). When* Solanum elaeagnifolium* was base treated the concentrations of metal ions adsorbed were 20.6 mg/g, 13.1 mg/g, 6.5 mg/g, 18.9 mg/g, 7.0 mg/g, 208 mg/g, and 2.2 mg/g of lead(II), copper(II), nickel(II), cadmium(II), zinc(II), chromium(III), and chromium(VI), respectively. Metal ion recovery from the bioadsorbent was done by using hydrochloric acid. This indicates that silverleaf nightshade can be used in treating the waste water containing toxic metal ions and it is cost-effective when compared to other methods implied previously [100].

### 3.15. Banana Peel

Fruit wastes like banana peel were used as bioadsorbent. It was best suited for copper removal from waste water. The banana peels were cut, washed, dried, and then grounded into powder for using it as a bioadsorbent. Some of the parameters like particle size, pH, temperature, and agitation speed, and contact time were studied to check the efficiency of copper adsorption onto banana peel. A pH of 6 was found to be optimum for the removal of copper ions. The adsorption capacity was found to be 27.78 mg/g. Studies also showed that one gram of banana peel can remove 7.97 mg of lead ions at a pH of 5.5 [101].

### 3.16. Leca

Leca or light expanded clay aggregate was used to remove heavy metals. Lead and cadmium adsorption capability of light expanded clay aggregate at different pH, contact time, and adsorbent concentration was studied. The effluents from paint industry are rich in lead and cadmium so those effluents were considered to perform batch studies. The adsorption was analyzed by varying the amount of leca in the range of 1, 2, 3, 4, 5, 6, 7, 8, 9, and 10 g/L. The amount of lead adsorption was found to be 93.75% on 10 g/L of leca at a pH of 7 and that for cadmium at the same conditions was 89.7%. The experimental data agreed well with Freundlich adsorption isotherm and the appropriate contact time was 1 to 2 hours. Thus leca can be used as a low cost adsorbent for treatment of heavy metals especially lead and cadmium from industrial waste water [102].

### 3.17. Papaya Seeds

The current methods of removing heavy metals are very costly so there is need for low cost adsorbent for the removal of metals like copper ions present in the aqueous solutions. The effect of pH, mixing rate, contact time, and adsorbate concentration was analyzed using batch studies to examine the rate of adsorption of copper ions on papaya seeds. The adsorption kinetic data were estimated by second-order and pseudo-first-order kinetics and the data fitted Langmuir and Freundlich isotherms very well. The maximum rate of adsorption of copper was seen when the pH was maintained at 6 and solution stirred at 350 rpm. 212 mg/g was the adsorption capacity of the papaya seeds. Chemisorptions process was denoted as the rate limiting step in the process of adsorption and it was found out by using the pseudo-second-order kinetic model as the data correlated well with this model. Finally these studies proved that papaya seed can be used as an effective adsorbent for the removal of heavy metals such as copper ions, from waste water [103].

### 3.18. Black Tea Waste

Studies showed that black tea waste can be used to remove heavy metals like zinc, cadmium, and cobalt from waste water. When the contact time was 180 minutes all the three metals were adsorbed. The adsorption capacity of heavy metals on tea waste mainly depends on the pH. An optimum pH of 6 is chosen for maximum adsorption of cobalt, cadmium, and zinc. Around 13.77 mg/g of Cd, 15.39 mg/g of Co, and 12.24 mg/g of Zn were adsorbed when only half gram of black tea waste was used. Experimental data fits well with the Freundlich and Langmuir isotherms. Hence tea waste can be used as one of the best and efficient adsorbents for waste water treatment [104].

### 3.19. Coffee Residues

The spent coffee grounds produced by instant coffee industry as a waste can be economically reused for the adsorption of toxic metal ions like copper, zinc, cadmium, and lead. The study was conducted with the aid of batch adsorption method in order to study the effect of parameters such as pH, metal ion concentration, adsorbent concentration, and temperature on metal adsorption. The best suited isotherm model was Langmuir adsorption isotherm in which the data was well fitted. Adsorption of metal ions to coffee residues was disturbed by flocculation at the adsorption site due to high density. So the formation of solid flocculent was treated by dispersant which showed increase in the maximum adsorption of metal ions. Using dilute acid solution the adsorption property was well maintained from the loss of change in the sites in column adsorption studies. It was reported that coffee grounds could remove the metal ions at the level of *μ*gL^−1^. Using potentiometric titration the acid base chemistry and stoichiometry of H^+^ ion were studied for tea leaves and coffee grounds. Hence the study concluded that tea and coffee residues can be used to remove trace metal ions from water [105].  Table 1 shows the specific metal adsorption capacity of various agricultural wastes where several authors evaluated the adsorption efficiency for various heavy metals by using different isotherm models which shows the interaction pattern between the adsorbate and adsorbent. An increase in temperature led to a decrease in the amount of metal adsorbed, regardless of the residue employed for adsorption process. The activation procedure was the same for many adsorbents but the coir fibers showed higher adsorption capacity (263 mg/g), followed by dal husk (96.05 mg/g), wheat bran (69 mg/g), pumpkin waste (68 mg/g), and coffee waste (63 mg/g). The least adsorption was shown by peanut hull (0.18–0.21 mg/g), grape bagasse (0.428 mg/g), walnut shell (1.33 mg/g), nut shell (1.47 mg/g), and exhausted coffee (1.42 mg/g). The surface area of any adsorbent is the deciding factor for its efficiency; that is, the higher the surface area, the higher the adsorption.

### 3.20. Sawdust

Studies were carried out on adsorption of chromium from the electroplating waste water [48]. Phosphate treated saw dust showed an increased adsorption of chromium when compared to the unmodified one. Ajmal et al., 1996, also reported that the adsorption of chromium depends on pH. A pH value of 2 and less than that was shown to be optimum for maximum removal of chromium from aqueous solution. Synthetic waste water and electroplating waste water containing about 50 mg/L of chromium were used in column process as well as in batch studies and these adsorbents showed 100% adsorption of chromium. 0.01 M NaOH was used to recover chromium ions. Activated carbon prepared from coconut tree saw dust was used to study the removal of chromium from aqueous solution through batch experiments [106]. Initial Cr(VI) concentration, carbon concentration, agitation time, and pH were the factors considered to study the adsorption process and the data was modeled using Langmuir and Freundlich adsorption isotherms. At an initial pH of 3.0 the adsorption capacity of the adsorbents with a particle size 125–250 *μ*m was found to be 3.46 mg/g using Langmuir isotherm. Acidic pH was optimum for maximum heavy metal removal [107].

Low cost adsorbents such as those discussed above are strongly recommended in industries for waste water treatment applications due to their local obtainability, technical viability, and engineering applicability. But before fermentation, acid-hydrolyzation of the agricultural wastes is done by breaking down the cellulose and lignin to produce bioethanol using microbes. If acid-hydrolysis is not done, it can result in lower yield. Other methods by which the biowastes could be treated are vacuum distillation, hydrotreatment, solvent extraction, thin film evaporation, and so forth. The contaminants can also be removed by using the right combination of heat and pressure at ambient conditions. In composting, enormous amount of readily organic matter which can be easily degraded is added to a contaminant that is further tailed by an aerobic incubation at a temperature ranging from 20 to 60°C. This becomes slightly labour intensive as the carbon and nitrogen ratios need to be adjusted frequently. This helps in converting the waste organic matter of used leaves, shells, husks, and peels to useful soil modifications by employing aerobic and anaerobic microbes which promotes the growth of certain bacteria, fungi, and actinomycetes. Land farming is another alternative method where the contaminated agricultural wastes or soil can be spread on fields to plow and fertilize the agricultural land which can be later redeveloped cost-effectively by planting heavy metal tolerant weeds and by using certain phytochelators. Heavy metals can also be precipitated as sulfates, carbonates, or hydroxide sulfides from the waste soil and water. Bioventing on the other hand promotes* in situ* biodegradation of biodegradable pollutants. This can also help in cleaning up of aquifers that are contaminated by solvents such as trichloroethylene which can be degraded only by cometabolic processes using enzymes. But some approaches can be costly which can be managed well by development of protein hydrolysates and by using activated carbon along with the agricultural wastes. The latest development in the green technology is biosorption of heavy metals by nonliving biomass of aquatic plant species such as* Hydrilla verticillata, Salvinia herzogii, Potamogeton lucens, Eichhornia crassipes, Ceratophyllum demersum*, and* Ludwigia stolonifera*. Another method employs microbes from the same site of pollution to accumulate heavy metals. These metals act as feed which is taken up for the microbial growth, metabolism, and living. The microbes release these heavy metals after a long time, but in a very lower concentration when compared to what they had initially absorbed. This process is known as bioaccumulation. The absorption of the toxic substances such as a metal is at a rate faster than that at which the substance is lost by catabolism and excretion. Other biological treatments using mixed microbial cultures and white rot fungi for decolorization of dyes are also common [108–111].

## 4. Conclusion

The existing technologies for waste water treatment have major problems such as costs involved in the construction of waste water treatment plants which are high; that is, they are uneconomical, consume lot of space, are commercially unattractive, and have disposal problems. Reports suggest that there is a bulk production of chemicals from various waste water treatment plants and requirement for a high electrical energy input. Handling of the dry sludge also becomes difficult and as well nature capacity to treat water remains unutilized. So there was a need for some alternative method which can overcome all these problems and treat the waste water in an appropriate way. Thus making use of bioadsorbents is an effective method to adsorb toxic heavy metals from effluents not polluting the ground water and at the same time utilizing the discarded open waste in the environment for a useful purpose of waste water treatment. This method not only requires minimal energy input, less labour, and low investment, but also proves to be cost-effective, but additional information about the pore size distribution of adsorbent, molecular size of metal ions, functional groups present on surface of adsorbent, initial pH, temperature, particle size of adsorbent, and so forth are critical details which influence the efficiency of adsorption processes. Several waste reduction programs should be held to reuse and recycle the wastes with an aim of zero waste production.

## Figures and Tables

**Table 1:** Comparison of adsorption capacity of various agricultural wastes.

Agricultural waste	Metal	Adsorption capacity (mg/g)	Reference
Wheat bran	Pb	69–87	[11]
Rice bran	Cu	27.81	[12]
Black gram husk	Pb, Cd, Zn, Cu, Ni	19.56–49.97	[13]
Dal husk	Cr(VI), Fe(III)	96.05, 66.63	[14]
Coffee waste	Pb	63	[15]
Exhausted coffee	Cr(VI)	1.42	[16]
Coffee husk	Cu	7.5	[17]
Tea residue	Cu/Pb	48–65	[18]
Almond shell	Pb	8.08, 28.18	[19]
Nut shell	Cr(VI)	1.47	[16]
Walnut shell	Cr(VI)	1.33	[16]
Chestnut shell	Cu	12.56	[20]
Peanut shell	Cu	21.25	[21]
Peanut hull	Cu/Pb	0.18, 0.21	[22]
Mango peel	Cu	46.09	[23]
Grape bagasse	Pb	0.428	[24]
Barley straw	Cu/Pb	4.64, 23.2	[19]
Saw dust	Cr(VI)	10.01, 16.05	[25, 26]
Coir fibers	Pb	263	[27]
Pumpkin waste	Cr(VI), Pb	68	[28]
Sugar beet pulp	Cu	31.4	[29]
Pea waste	Cr(VI)	21.2	[30]
